# Net, skin and flatten, ImageJ plugin tool for extracting surface profiles from curved 3D objects

**DOI:** 10.17912/micropub.biology.000292

**Published:** 2020-08-19

**Authors:** Housei Wada, Shigeo Hayashi

**Affiliations:** 1 RIKEN Center for Biosystems Dynamics Research

**Figure 1. Surface flattening procedure:**
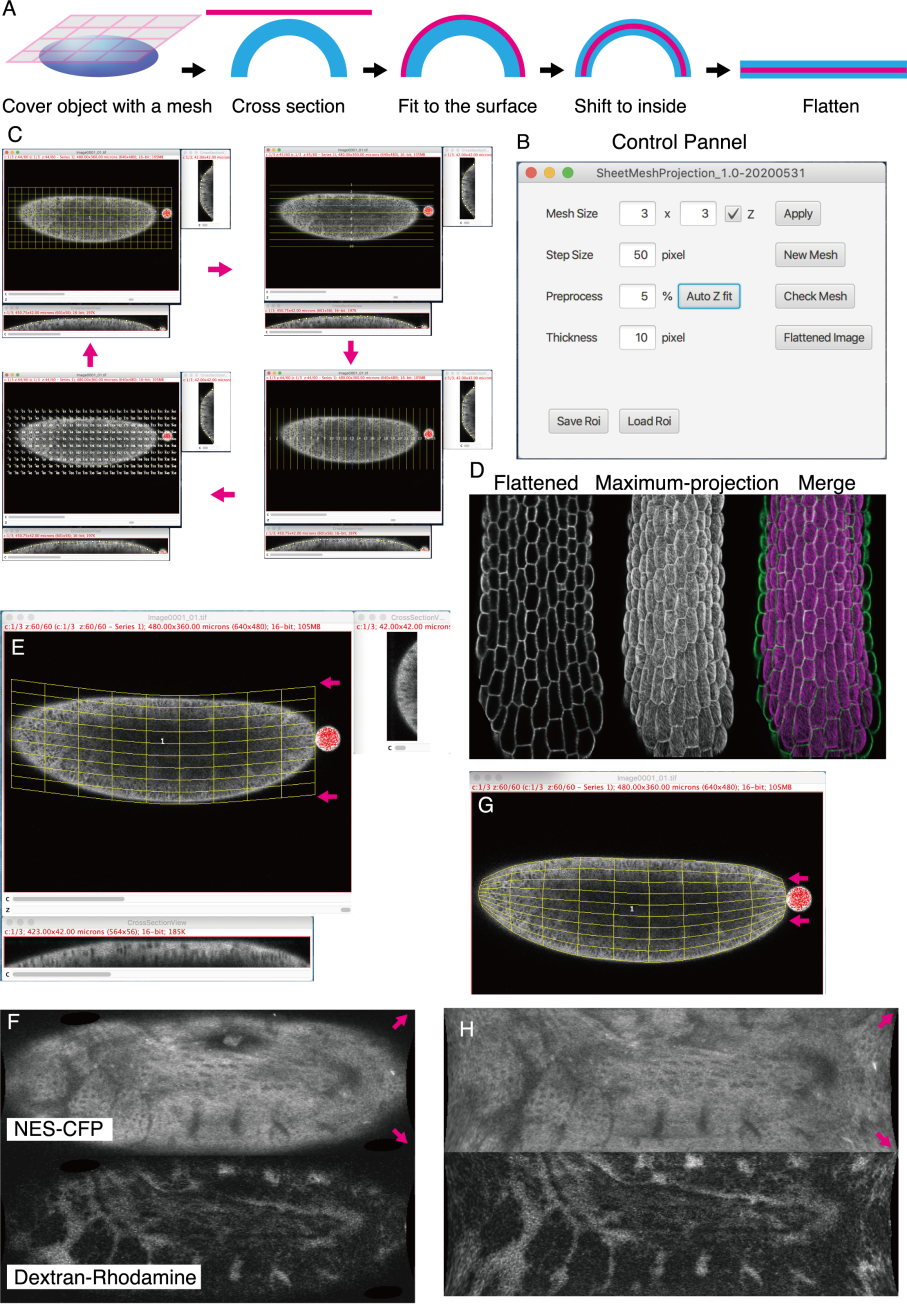
(A) Mesh is placed over the object and mesh lines are fitted to the surface. Then the lines are shifted inside and the object is flattened along this line. (B) User interface panel. (C) The appearance of the mesh changes to lines and cross point each time you press the space key. Lines can be selected by clicking the number and its position can be adjusted in x-y, y-z, or x-z windows. (D) Hypocotyl image stack flattened by SheetMeshprojection (left), maximum projection (middle), and overlay (right). (E-H) *Drosophila* embryo ubiquitously expressing nuclear export signal (NES) fused to CFP (Ogura et. al., 2018) was injected with dextran-RITC into the perivitelline space, which remains as a drop at the posterior end (red). Flattening with a mesh of rectangle (E, F) or latitude-longitude coordinate (G, H). Polar regions are expanded in the latter.

## Description

Optical cross-sectioning of whole embryos with advanced fluorescent microscopes such as confocal laser scanning microscopes or light-sheet microscopes has been extensively used in developmental biology. The maximum projection of markers localized to the object surface is widely used to reconstruct surface profiles (Erguvan *et al.*, 2019). However, this technique is effective only to the surface parallel to the plane of sections, and area profiles of diagonal surfaces, such as the margin of embryos, are under-represented. Tools for tracing curved surfaces have been developed (de Reuille *et al.*, 2015; Heemskerk & Streichan, 2015; Viktorinová *et al.*, 2019), but their usage is not easy for novice users. A simple and easy-to-use tool with basic surface extraction function for users of the most widely used image analysis platform, ImageJ (https://imagej.nih.gov) is anticipated. For this reason, we developed a highly versatile ImageJ plugin “SheetMeshProjection” for extracting curved surfaces with user-defined thickness and creating expanded 2D images.

A step-by-step manual is provided in the plugin package. Unlike conventional projection software that recognizes the surfaces pixel by pixel, SheetMeshProjection uses a coarsely spaced mesh to capture the surfaces of objects like casting a net (Fig. 1A). If necessary, each line of the mesh is manually corrected to fit the surfaces in x-z or y-z cross-sectional view, and lines can be adjusted to longitude to cover spherical objects (Fig. 1G, H). Mesh line position can be moved to avoid positions of hairs and other small appendages that potentially confuse surface tracking. Mesh density can be increased later to refine tracking (Fig. 1B). After fitting the mesh line position, the surfaces of user-defined thickness is extracted and converted to a flattened object, like skinned fur rugs.

Images extracted in this way provide more faithful representations of the surface of 3D objects with acute curvature, especially at the margin (Fig. 1D). It should be noted that the appearance of flattened images varies if location and spacing of mesh lines are changed (Fig. 1E-H) and quantitative information (length and area) of the extended surfaces may not be preserved. Flattened images should be regarded as qualitative representations of the object surfaces. For the precise acquisition of 2D-projected surface images, more sophisticated software is available (Schmid *et al.*, 2013), although it is limited to spherical objects. Flattened surface images of whole embryos or organs with convex surfacesmay be used to help analysis of cell counting and cell neighborhood analysis and should help studies of complex cell composition of 3D biological objects.

## Reagents

Software Availability: The plugin is available at Fiji update site (https://sites.imagej.net/SheetMeshProjection/). Full package of plugins, instruction manual, and a test image is available from the authors’ home page https://signaling.riken.jp/en/en-tools/imagej/1743/). Source code and documentation (ver. 1.0-20200721, MIT) are available on GitHub (https://github.com/Wada-H/SheetMeshProjection). An archived version of this data is available on Caltech Data: DOI 10.22002/D1.1606.

Drosophila strain: CFP fluorescence of the *pubi-ekarev-nes^58A^* FRET sensor strain (Ogura et. al., 2018) was shown as the NES-CFP image (Fig. 1F).
